# Dynamic-weighing grain mass flow sensing for reliable in-field yield monitoring in combine harvesters

**DOI:** 10.3389/fpls.2026.1825426

**Published:** 2026-05-19

**Authors:** Feng Wang, Zhijun Meng, Liping Chen, Wuchang Qin, Fei Dai, Fengwei Zhang

**Affiliations:** 1 College of Mechanical and Electrical Engineering, Gansu Agricultural University, Lanzhou, China; 2 Research Center of Intelligent Equipment, Beijing Academy of Agriculture and Forestry Sciences, Beijing, China

**Keywords:** cumulative mass closure, inclined screw conveyor, nonstationary disturbance, residence time estimation, validity gating

## Abstract

The accuracy of on-board yield monitoring for combine harvesters is limited by the difficulty of acquiring high-fidelity mass flow signals and achieving reliable cumulative integration under complex vibration and varying operating conditions. To address this challenge, this study developed a weighing-based on-board yield monitoring system (DW-ORMS), in which a flow-collecting weighing-type grain mass flow sensor (C-GMFS) served as the core sensing unit. The C-GMFS enables stable weighing observation in confined installation spaces through mechanical decoupling and a single force-transmission path. At the system level, a state-aware gating strategy and trusted update mechanism were introduced to improve the reliability of output and cumulative mass estimation during unsteady operating phases. Based on field-measured disturbance characteristics, a parameterized disturbance model was established and used to identify the valid operating window and fix the gating parameter range. Simulation results showed that the proposed method achieved band-limited interference suppression of *A_band_*≥17.73 dB under strong disturbances while maintaining trend fidelity of *PRR_trend_*≥41.73% and low processing delay. Field harvesting experiments were conducted at working speeds of 2–8 km·h^−^¹. The cumulative strip mass showed excellent agreement with manual weighing references, with *R*²=0.973 and *RMSE* = 0.84 kg, and the strip-level mass closure error remained within CE ≤ 5% (N = 35). No monotonic drift in error was observed across working-speed groups. These results demonstrate that the DW-ORMS provides a deployable and traceable solution for high-confidence on-board yield monitoring of combine harvesters under unsteady field conditions.

## 1 Introduction

The core of smart agriculture lies in the precise perception and closed-loop regulation of agricultural production processes through the deep integration of information technology and physical systems (Wang, 1999; Zhao et al., 2003; Montalvo et al., 2025). In combine harvesting operations, grain mass flow is widely recognized as a key input for yield monitoring systems because it directly supports yield mapping, variable-rate operations, and adaptive control of harvester feed state and load variations (Monteiro et al., 2021; Longchamps et al., 2022; Chung et al., 2016; Wang et al., 2021; Gauci et al., 2025; Sun et al., 2022; Liang et al., 2024; Sun et al., 2025). More broadly, recent precision-agriculture studies have highlighted the increasing importance of intelligent and interpretable sensing frameworks for field-level monitoring and decision support (Goyal et al., 2025; Malik et al., 2026).

However, stable grain mass flow monitoring remains difficult under complex field conditions. Electromagnetic interference, temperature and humidity fluctuations, feed-rate variation, and field spatial heterogeneity can introduce measurement uncertainty and strongly nonstationary signal characteristics, thereby increasing the requirements for sensing reliability, dynamic response, and disturbance rejection (Loghavi et al., 2008; Yang et al., 2024). These challenges are particularly severe at the discharge end of an inclined screw conveyor, where the grain stream is often intermittent and uneven and is further coupled with whole-machine vibration and limited installation space, causing baseline drift, transient signal distortion, and a shortened effective observation window (Chen et al., 2022).

Existing methods for grain mass flow sensing can generally be classified into impact-based, volumetric, and weighing-based approaches (Arslan et al., 2000; Chung et al., 2016; Jin et al., 2020; Wang et al., 2021). Impact-based methods are sensitive to vibration, baseline drift, and installation conditions (Zhou and Liu, 2006; Xiong et al., 2018; Liu et al., 2022; Chai et al., 2024; Fang et al., 2024). Volumetric methods are susceptible to dust, humidity, material adhesion, and bulk-density conversion errors, which limit long-term stability and cross-crop adaptability (Fu et al., 2017; An et al., 2017; Jin et al., 2022; Yin et al., 2024). Weighing-based methods have the inherent advantage of directly measuring mass, but in screw conveyor scenarios they still suffer from dynamic distortion caused by vibration, grain-flow impact, and spatial constraints (Wagner and Schrock, 1989; Zhang et al., 2010; Wang et al., 2026). In essence, these limitations arise because dynamic metrology problems are often treated as static calibration problems; therefore, systematic optimization of measurement boundaries, force-transfer paths, and disturbance suppression remains essential for practical weighing-based grain mass flow monitoring.

Compared with horizontal or vertical screw conveyor configurations, the inclined discharge end exhibits stronger flow intermittency, more pronounced impact effects, and tighter spatial constraints (Schuster, unpublished; Fuchs et al., 2007; Moser et al., 2008; Nadimi et al., 2023). Although this makes deployment more difficult, it also offers the advantages of direct mass observation and cumulative mass-closure validation. At the same time, existing studies have mainly focused on individual sensor structures or standalone calibration methods, and coordinated design across the full chain of sensing, acquisition, processing, data management, and validity verification is still insufficient (Jin et al., 2020; Wang et al., 2021). Because inclined screw conveyors are widely adopted in large-feed-rate combine harvesters to improve grain-bin filling, space utilization, and machine balance, the discharge end becomes a representative and practically important location for grain mass flow monitoring (Fuchs et al., 2007; Nadimi et al., 2023; Moser et al., 2008).

Therefore, the primary challenge addressed in this study is to achieve accurate and stable grain mass flow monitoring at the discharge end of an inclined screw conveyor. Three unresolved technical bottlenecks remain: inadequate measurement boundaries and force-transfer paths, insufficient quantification and parameterization of disturbance mechanisms, and the lack of online validity management during operation. Compared with prior weighing/dynamic-weighing methods, such as grain-tank weighing and retrofitted dynamic-weighing structures in grain conveying systems, the present study offers three main innovations. First, a flow-collecting dynamic-weighing scheme is proposed for the inclined discharge end to establish a reproducible measurement boundary and a stable force-transfer path without major modifications to the original conveying channel. Second, a physically interpretable mass-flow model is developed by introducing an equivalent residence-time variable derived from the time-domain characteristics of impact events in the weighing signal, thereby reducing dependence on empirical velocity coefficients and purely calibration-based models. Third, a state-recognition and gated-integration strategy is introduced so that cumulative integration is performed only during valid operating stages, mitigating baseline drift and abnormal accumulation under unsteady conditions and improving the stability of closure error (CE).

To address these issues, this study develops a flow-collecting weighing-type grain mass flow sensor (C-GMFS), constructs an on-board real-time yield monitoring system (DW-ORMS), and establishes a verification chain of field feature extraction, parameterized disturbance simulation, on-board implementation, and field mass-closure validation. The main work of this study is summarized as follows.

1. A weighing-based mass-flow monitoring scheme is proposed for the inclined screw conveyor discharge end under the coupled constraints of limited space, intermittent discharge, and composite vibration, together with its engineering implementation path.
2. The C-GMFS is developed as the core sensing unit to achieve non-destructive retrofit and online weighing observation through a reproducible flow-collecting measurement boundary, a mechanically decoupled stable force-transfer path, and a detachable installation structure.
3. A single-channel weighing-based method combining field feature extraction, parameterized disturbance simulation, and parameter fixation is established to determine disturbance boundaries, operational-state thresholds, and signal validity gating rules for deployable online processing.
4. The DW-ORMS is implemented and systematically validated under field conditions using strip-scale yield closure consistency as the core metric, demonstrating its effectiveness, repeatability, and engineering applicability for continuous and stable weighing-based grain mass flow monitoring.

## 2 Materials and methods

### 2.1 Overall architecture of DW-ORMS

**Figure 1:**
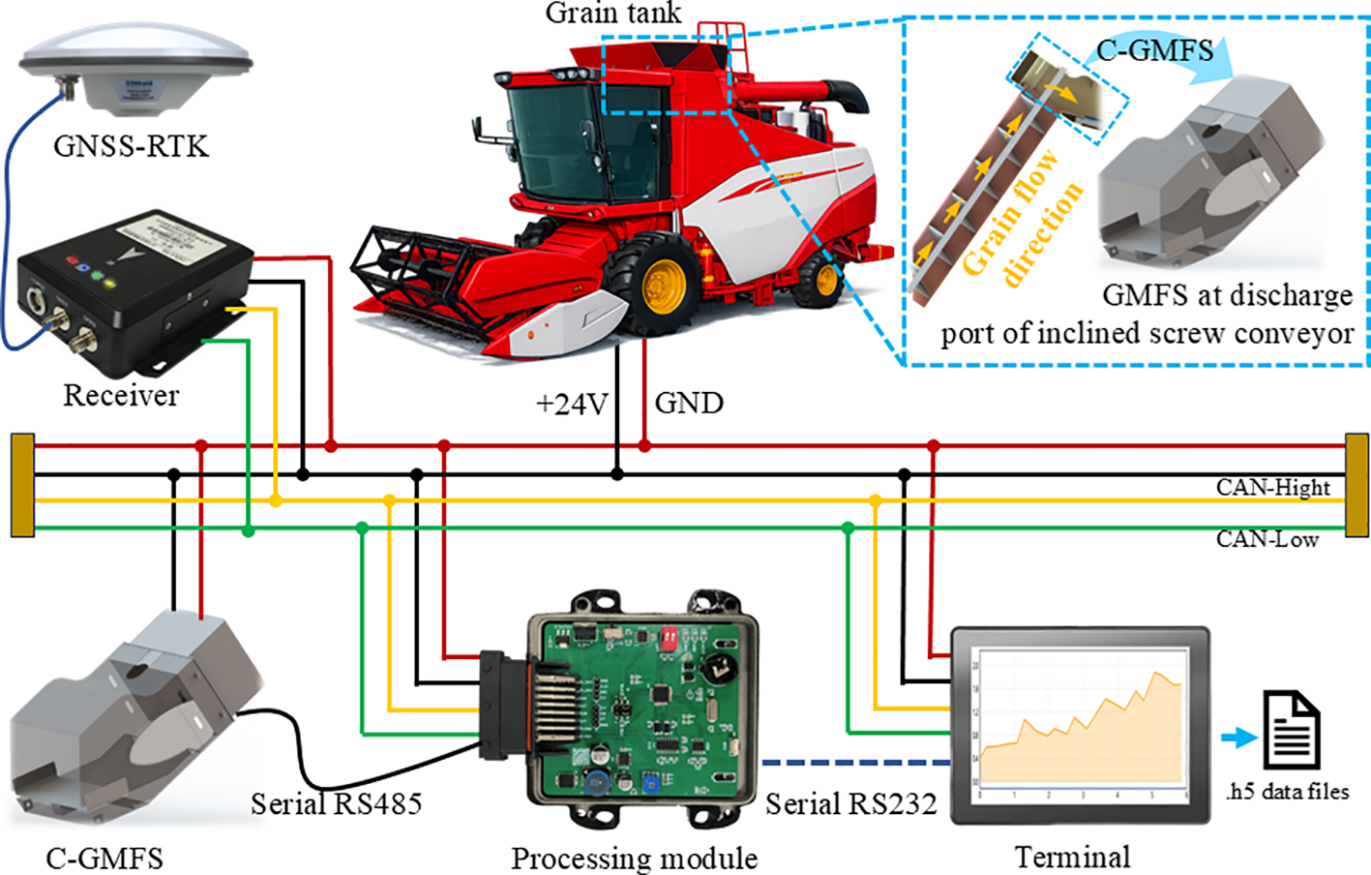
Overall architecture of the DW-ORMS, including the C-GMFS, the data acquisition and processing unit, and the on-board terminal.

To achieve stable acquisition of grain mass flow and cumulative mass under field operating conditions of a combine harvester, this study constructed the DW-ORMS. As shown in **Figure 1**, the system consists of the C-GMFS, a data acquisition and processing unit, and an on-board terminal. A core workflow is adopted for system operation: high-frequency sampling of weighing signals, online preprocessing with status and validity management, real-time mass flow calculation, and cumulative mass recording. It delivers stable instantaneous mass flow and cumulative net grain mass output even under complex field conditions like coupled vibration or intermittent discharge. Moreover, it provides reliable data support for consistency evaluation of measurement results using the cumulative mass closure method.

**Figure 2:**
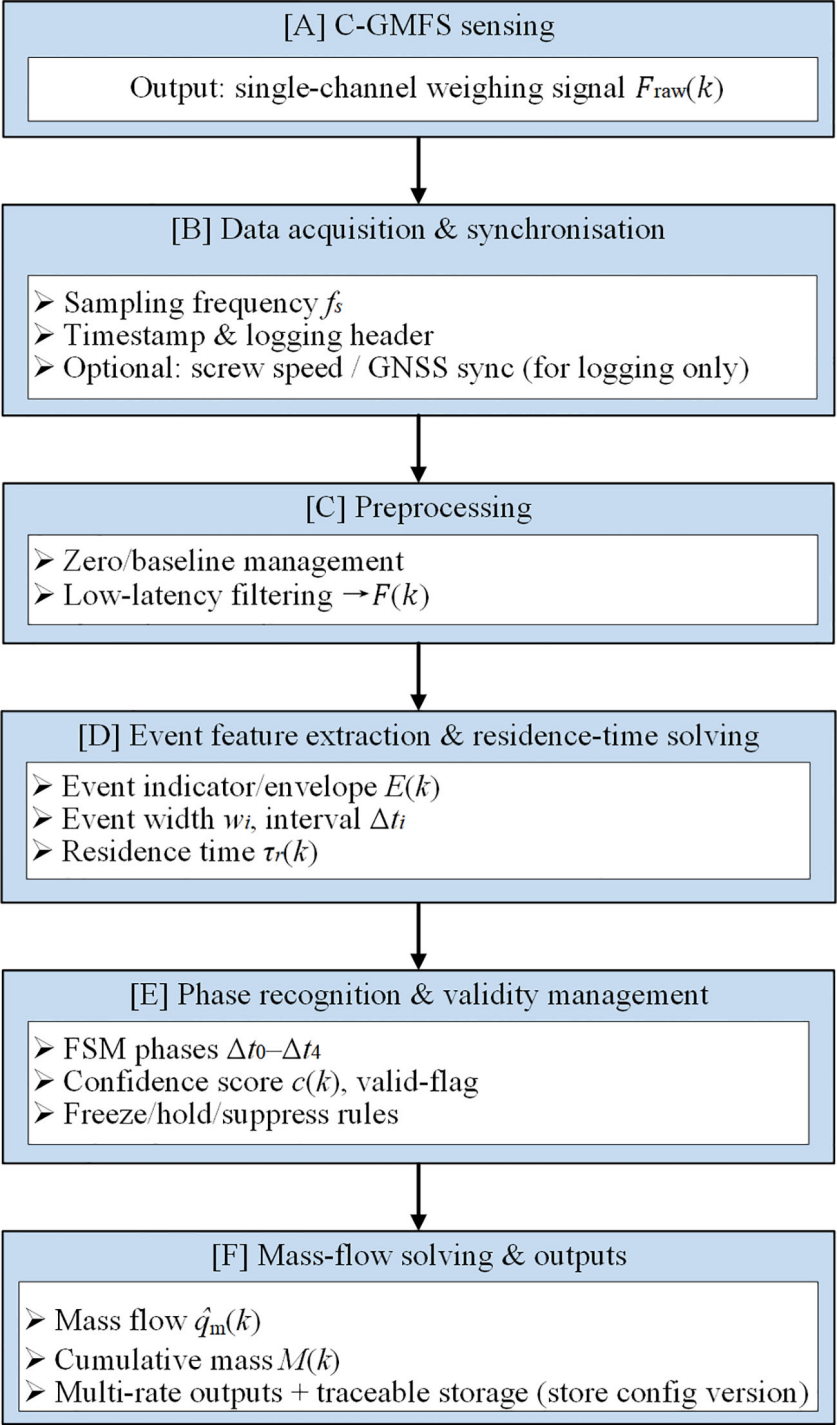
System block diagram of the DW-ORMS, showing the signal path from weighing acquisition to online processing and output.

**Figure 3:**
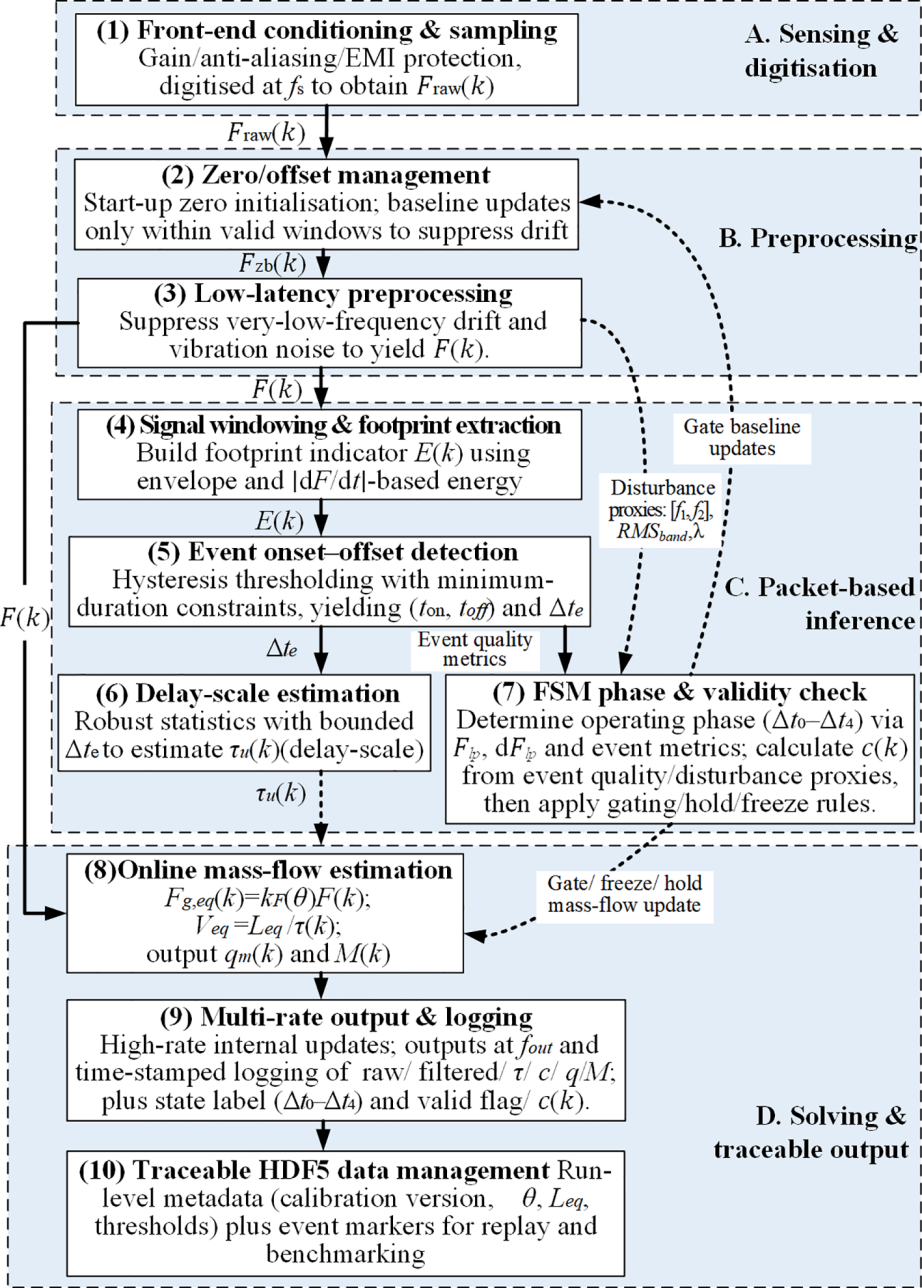
Online signal processing and data handling pipeline for residence-time-based mass flow rate solving using a single channel weighing signal. The raw weighing signal is preprocessed and filtered, followed by operational phase recognition and validity gating. During valid windows, the residence time *τ_r_* is estimated from time-domain event features and used for mass flow reconstruction; during invalid windows, the output is withheld to avoid contaminated updates. The final outputs include instantaneous mass flow rate and cumulative mass for strip-level closure validation.

**Figure 4:**
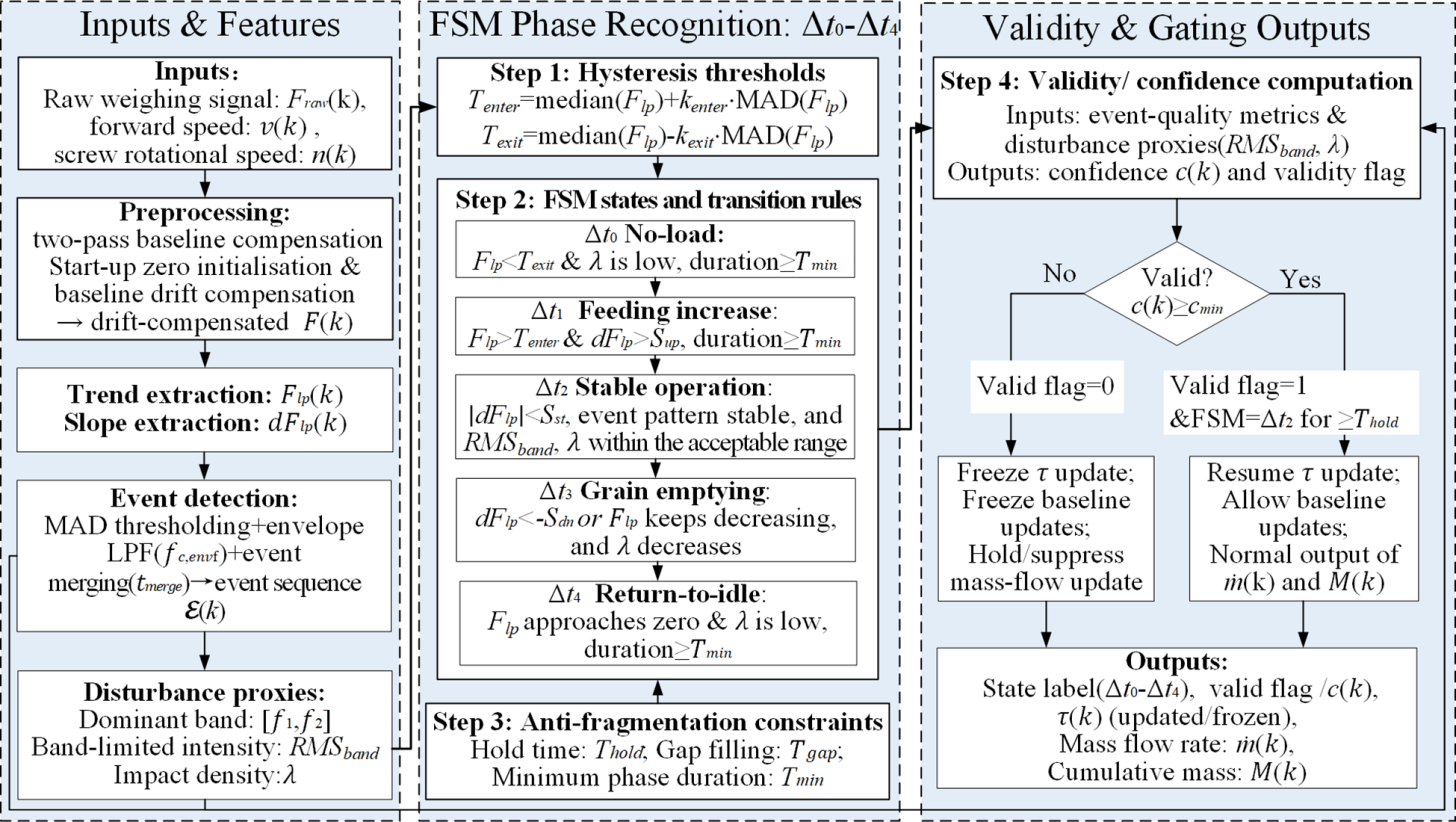
Rule-based FSM for phase recognition (Δ*t*_0_–Δ*t*_4_) and validity gating.

The data acquisition and processing unit is designed to perform signal conditioning and high-frequency sampling of the raw weighing signals. It also performs zero and baseline management, signal preprocessing, operational phase recognition, and validity gatekeeping, as well as mass flow calculation and cumulative integration in the on-board embedded terminal. Key monitoring parameters are output in real time with configurable update rates. The on-board terminal is designed to realize parameter configuration, operation status monitoring, measurement result visualization, and data management. The monitoring data is organized with unified timestamps and stored in HDF5 format files following a hierarchical structure: raw signal - processed signal - event and status markers - calculation results. This facilitates subsequent calibration traceability and offline error analysis. Two standardized data output modes are designed for the system: a serial port and a CAN bus. The serial port is mainly used for system calibration, debugging, and data playback, while the CAN bus is used for integration with the combine harvester control system to provide interface support for subsequent functional expansion. A concise system-level block diagram of the DW-ORMS is provided in **Figure 2** to summarize the hardware composition and the end-to-end data flow from sensing and acquisition to online processing, output, and traceable storage. The detailed online signal-processing pipeline and the FSM-based decision logic are further presented in **Figures 3** and **4**, respectively.

### 2.2 Field setup and validation protocol

To validate the monitoring performance of the DW-ORMS under real harvesting conditions, this study conducted strip field validation tests. The cumulative mass closure error and linear consistency of the system were evaluated under different forward speeds and non-uniform field spatial backgrounds.

#### 2.2.1 Field setup and operating conditions

**Figure 5:**
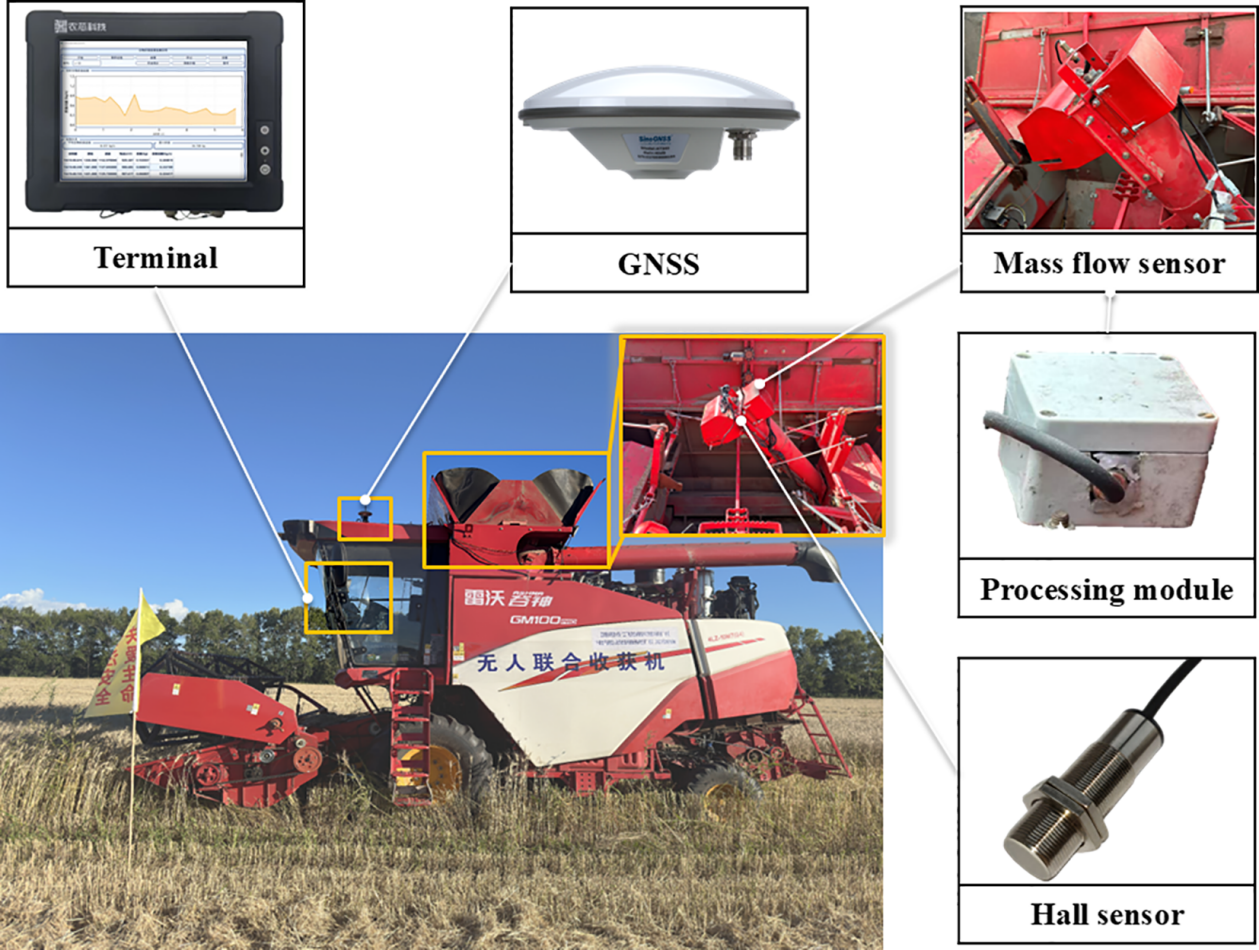
Field test platform.

The field experiment was conducted in August 2025 at Hongxing Farm, Heihe City, Heilongjiang Province (48.1282°N, 126.9649°E), with Longmai 35 spring wheat as the test crop. The grain moisture content during the harvest period was 12.5%–15.16%, measured by a portable moisture meter (Model 1DS–1G, Tianjin Lookout Photoelectric Technology Co., Ltd., China). The test platform was a GM100 Pro combine harvester (4LZ-10M7 (G4), Weichai Lovol Intelligent Agricultural Technology Co., Ltd., China) (**Figure 5**), whose grain conveying system is equipped with an inclined screw conveyor. According to the structural implementation scheme, the C-GMFS was installed at the discharge end of the inclined screw conveyor, achieving non-invasive online weighing without altering the original main conveying structure and operation process. The consistency of installation posture, force transmission conditions, and calibration configuration was maintained to minimize system deviation caused by assembly differences.

To obtain high-precision spatiotemporal data, the DW-ORMS was integrated with the harvester’s RTK-GNSS terminal, which received NMEA messages (e.g., GGA, RMC) at 10 Hz to acquire position, ground speed, and heading data. The system’s sampling parameters were configured as follows: weighing signal at 1000 Hz, screw conveyor rotational speed signal at 50 Hz, and GNSS at 10 Hz. All channel data were timestamped by a unified system clock to facilitate subsequent identification of valid operational intervals and data alignment.

The strip operation design covered the forward speed range of 2-8 km·h^−^¹, forming a typical low-to-high feed rate gradient. This forward speed range was selected because it generally covers the conventional effective operating speeds of rice–wheat combine harvesters under practical harvesting conditions, spanning low, medium, and relatively high working speeds. Within this interval, the harvester can usually maintain acceptable feeding continuity, threshing and cleaning performance, and harvesting quality. When the forward speed is lower than 2 km·h^−^¹, harvesting efficiency decreases markedly and the energy consumption per unit area increases; meanwhile, the feeding rate may become too low to maintain a stable material flow or material layer in the threshing and cleaning units. In contrast, when the forward speed exceeds 8 km·h^−^¹, the feeding rate may approach or even exceed the design capacity of the harvester, thereby increasing threshing drum load and thickening the material layer on the cleaning shoe, which may lead to higher grain breakage, incomplete threshing, increased cleaning loss, and a higher risk of material blockage. Therefore, the 2–8 km·h^−^¹ range was used as the main validation window in this study. A total of 36 strips were completed in two experimental fields. The data quality control process included: GNSS fixed solution continuity check, weighing signal saturation and abnormal drift check, communication packet loss check, and grain cleaning and emptying check at the end of operation. Finally, one abnormal strip was excluded, and 35 valid strips were retained for performance evaluation.

#### 2.2.2 Reference mass acquisition and validation protocol

**Figure 6:**
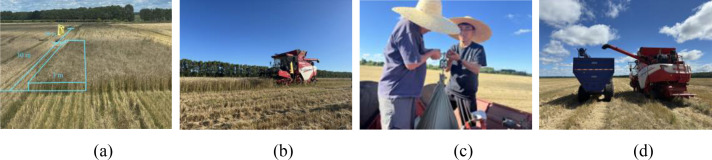
Strip-trial workflow and reference grain-mass acquisition for field mass-closure validation: **(a)** strip-trial division; **(b)** harvesting operation process; **(c)** manual weighing and data recording; **(d)** final grain unloading.

The net grain weight of each strip served as the ground truth for closure validation. During harvesting, clean grain was collected segmentally in woven bags at the discharge outlet of the C-GMFS. After completing a strip, the bags were weighed using an electronic hanging scale (WH-C100, range 0–150 kg, resolution 0.1 kg, Guangzhou Weiheng Electronic Technology Co., Ltd., CN) to obtain the reference mass *M_i_*. Based on a pilot test, the strip length was set to 30 m, which ensured the representativeness of each run while enabling continuous full-strip collection of cleaned grain to obtain a reliable cumulative-mass ground truth. Although losses in upstream threshing and cleaning processes were not considered, this outlet-based protocol provided a direct and traceable ground truth for sensor-level cumulative mass-closure validation because it reflected the total grain mass passing through the monitoring region. The grain mass acquisition and data collection procedure is illustrated in **Figure 6**, including strip division (**Figure 6a**), field harvesting and grain collection (**Figure 6b**), manual weighing and recording (**Figure 6c**), and final unloading (**Figure 6d**).

The online verification followed the unified process of “static calibration coefficient consistency, online solution output, and strip closure matching.” First, the C-GMFS raw voltage readings were converted into an equivalent load time series based on the static calibration curve, with the conversion logic and parameter version kept consistent between the embedded system and offline recalculation. Second, the DW-ORMS performed online preprocessing, validity management, and real-time mass flow calculation, outputting the instantaneous mass flow $\hat{q}$_m_(*t*) and integrating it (within the validity-gated window) to obtain the cumulative mass $\hat{M}$(*t*).

To evaluate strip-level closure, the effective harvesting interval [*t_s_*,*_i_*,*t_e_*,*_i_*] for each strip was identified based on GNSS trajectories and mass flow curves. Non-operational phases such as idling, turning, and waiting were excluded, and the instantaneous mass flow was integrated within the effective harvesting interval to obtain the system-estimated cumulative strip mass, as shown in Equation 1.

(1) $${\hat{M}}_{i}=\int_{t_{s,}i}^{t_{e,}i}{\hat{q}}_{m}(t)dt$$

The evaluation dataset was constructed by pairing the system estimates with the reference truth values ( $\hat{M}$*_i_*, *M_i_*), where $\hat{M}$*_i_* represents the system-estimated cumulative mass of the *i*-th strip and *M_i_* represents the reference truth value.

#### 2.2.3 Metrics and statistical analysis

Given the difficulty in obtaining real-time truth values of instantaneous mass flow under field conditions, this study used strip-level mass-closure consistency as the core evaluation objective. The closure error (CE) was defined as the signed relative error of the cumulative strip mass, as shown in Equation 2:

(2) $$CE=\frac{{\hat{M}}_{i}-M_{i}}{M_{i}}\times 100\%$$

Meanwhile, overall accuracy and linear consistency were evaluated using mean absolute error (*MAE*), mean relative error (*MRE*), root mean square error (*RMSE*), and coefficient of determination (*R*^2^). All overall metrics were calculated based on the 35 valid strips, and the distributions of *CE* and *MRE* were also reported. To analyse the effect of forward speed on closure performance, the valid strips were grouped by realised average forward speed into six bins: 2–3, 3–4, 4–5, 5–6, 6–7, and 7–8 km·h^−^¹. For each group, *CE* and related error metrics were analysed separately, and uncertainty was described using both interval-based and robust statistics, including the 95% confidence interval of the mean, the median, the interquartile range (*IQR*), and the mean absolute error (*MAE*). Taken together, these statistics provide a more complete description of grouped closure performance under different feeding levels.

### 2.3 Field disturbance characterization and parameterized simulation framework

To support the design of the online state-recognition and validity-gating strategy, field disturbances were first characterized from measured weighing signals, and the resulting statistics were then used to define the admissible parameter ranges for the parameterized simulation framework.

#### 2.3.1 Field disturbance characteristics statistics

Based on the raw weighing signals collected from field tests, zero-point correction and basic preprocessing were performed first. Subsequently, the disturbance characteristics of the inclined screw conveyor were statistically analyzed. The statistical indicators included dominant frequency band, band-limited vibration intensity, and impact density. The dominant frequency band was estimated using the Welch power spectrum density (PSD) method. The main peak frequency *f_p_* was identified within the preset low-frequency range, and the energy concentration interval [*f*_1_,*f*_2_] was determined by the narrowest frequency band with 80% cumulative energy, which characterizes the primary vibration components under different forward speeds. The vibration intensity was characterized by the *RMS_band_* of the target frequency band, which was calculated via spectral integration of the weighing signal after removing the quasi-static component within the frequency range [*f*_1_,*f*_2_]. The impact density *λ* was characterized by the peak arrival rate: the MAD-robust threshold was applied to the preprocessed signal for peak detection, and the number of impact peaks meeting the threshold within a unit time was counted.

The resulting global P10–P90 intervals used to define the admissible parameter ranges for the simulation framework are summarized in **Table 1**.

**Table 1:** Global P10–P90 ranges of field disturbance statistics used for parameterized simulation.

Module	Parameter	Symbol	Fixed value/Interval	Unit
Sampling	Sampling frequency	*f*_s_	1000	Hz
Operation state recognition (Δ*t*_0_-Δ*t*_4_)	Load trend cutoff frequency	*f*_c_	0.3 (0.2-0.5)	Hz
	Slope smoothing cutoff frequency	*f*_c_’	0.5 (0.3-1.0)	Hz
	Hysteresis hold time	*T_hold_*	2 (1-3)	s
Disturbance modeling parameters	Dominant disturbance frequency band	[*f*_1_,*f*_2_]	[0.73, 17.82]	Hz
	Disturbance intensity (band-limited rms)	*RMS_band_*	[32.02, 52.34]	N
	Impact density	*λ*	[1.35, 3.60]	s^-1^
	Delay scale	*τ_u_*	[1.09, 3.11]	s
Event detection parameters	Mad coefficient	*k_mad_*	2.3	–
	Envelope low-pass cutoff frequency	*f_c,env_*	15	Hz
	Event merging interval	*t_merge_*	0.08	s

[*f*_1_,*f*_2_], *RMS_band_*, *λ*, *τ_u_* denote global P10-P90 intervals across all field runs. Thresholds *T_enter_*, *T_exit_*, *S_up_*, *S_st_* and *S_dn_* are adaptively derived from median–MAD robust statistics of *F_lp_* and *dF_lp_*. Two-pass baseline correction, gap-filling, and minimum-duration constraints are used to ensure temporally continuous and physically consistent phase segmentation.

#### 2.3.2 Parametric disturbance simulation model

Based on the above statistical results, a parameterized disturbance simulation model was constructed for offline analysis. The simulation input was composed of ideal weighing load, continuous vibration component, and random impact component. The continuous vibration component was generated by a band-limited stochastic process with a frequency band of [*f*_1_,*f*_2_], and its amplitude was matched to the field-measured *RMS_band_*. The random impact component was described by a Poisson process to model the arrival behavior, with the impact arrival rate given by *λ*. The response to a single impact was represented by an exponentially decaying impulse. A simulation dataset covering different disturbance levels and parameter combinations was formed through parameter scanning (**Table 1**).

### 2.4 Dynamic model and signal processing for grain mass flow monitoring

The DW-ORMS relies on dynamic weighing signals from the C-GMFS to calculate grain mass flow in real time. Previous studies have developed and validated dynamic weighing models for vertical and horizontal screw conveyors (Wang et al., 2026). However, when applied directly to the discharge end of inclined screw conveyors—characterized by spatial constraints, intermittent discharge, and strong coupled vibrations—these models may encounter issues such as exacerbated calculation fluctuations, baseline drift, and a shortened effective observation window. To address this, this study built upon the existing preliminary dynamic weighing model, and leveraged the stable measurement boundary established by the C-GMFS’s collecting-type structure and its well-defined mechanically decoupled force transmission path. An online mass flow calculation method tailored to the inclined screw conveyor scenario was thus constructed, to improve the adaptability of the mass flow model to this harsh operating scenario.

During combine harvester operation, the signals acquired by the C-GMFS are often coupled with multi-source disturbances, including whole harvester coupled vibration, feed rate fluctuations, and discharge impacts. To mitigate the effects of these disturbances on model accuracy, a low-latency signal and data processing chain was designed in this study. On the mechanical side, the collector structure and force transmission path were optimized to suppress coupled disturbances. On the digital side, online preprocessing, filtering, and hierarchical multi-rate computation were implemented, combined with static calibration, online adaptive compensation, and zero-point management under operating conditions. This integrated processing chain is designed to improve the stability and traceability of mass flow output in complex field environments, thereby providing a reliable data foundation for subsequent operational state identification and online validity management. For readability, the main variables and symbols repeatedly used in the modelling and online processing are summarised in **Table 2**. Less frequently used auxiliary parameters are defined at their first appearance in the text.

**Table 2:** Nomenclature and symbols used in the modelling and online processing.

Symbol	Definition	Unit
$\hat{q}$_m_(*k*)	System measures grain mass flow rate	kg·s^-1^
*M*(*k*)	Cumulative mass obtained by gated integration of $\hat{q}$_m_	kg
*F*_raw_(*k*)	Raw weighing signal from the load cell	N
*F*(*k*)	Conditioned/filtered weighing signal used for feature extraction	N
*F_g,eq_*(*k*)	Equivalent gravity component reconstructed for the measurement region	N
*k_F_*(*θ*)	Attitude correction coefficient	–
*θ*	Fixed installation angle used in attitude correction	rad
*L_eq_*	Equivalent length of the measurement region	m
*τ_r_*(*k*)	Equivalent residence time solved from event footprints	s
*τ_u_*	Trusted update time scale used for online update management	s
*E*(*k*)	Event indicator/envelope feature for event detection	–
*w_i_*	Width (duration) of the *i*-th detected event	s
Δ*t_i_*	Interval between successive detected events	s
*CV_w_*	Coefficient of variation of event widths {*w_i_*}	–
*CV*_Δ_ *_t_*	Coefficient of variation of event intervals {Δ*t_i_*}	–
*c*(*k*)	Confidence score for validity assessment	–
*valid-flag*	Validity indicator (1 valid/0 invalid)	–
Δ*t*_0_–Δ*t*_4_	FSM phases: no-load, feed increase, stable, emptying, return-to-idle	–
*f*_s_	Sampling frequency	Hz
[*f*_1_, *f*_2_]	Dominant disturbance frequency band	Hz
*RMS_band_*	Disturbance intensity (band-limited rms)	N
*λ*	Impact density	s^-1^

#### 2.4.1 Grain mass flow calculation model

To extend the dynamic weighing mass flow model to the collecting-type weighing scenario at the discharge end of the inclined screw conveyor, this study derived an online solution model based on the principle of “instantaneous grain mass in the measurement zone – equivalent residence time”.

Given that the equivalent mass of grains in the measurement zone can be characterized by its vertical equivalent gravitational component, the instantaneous mass flow rate *q_m_*(*t*) can be expressed as:

(3) $$q_{m}(t)=\frac{F_{\text{g,eg}}(t)}{g}\cdot \frac{V_{eg}(t)}{L_{eq}}$$

where, *q_m_*(*t*) denotes the instantaneous mass flow rate, kg·s^−^¹; *F_g,eq_*(*t*) represents the equivalent gravitational component of grain in the measurement zone, N; *V_eq_*(*t*) is the equivalent average flow velocity, m·s^−^¹; *L_eq_* is the equivalent length of the measurement zone, m; and g is the gravitational acceleration, m·s^−^².

**Figure 7:**
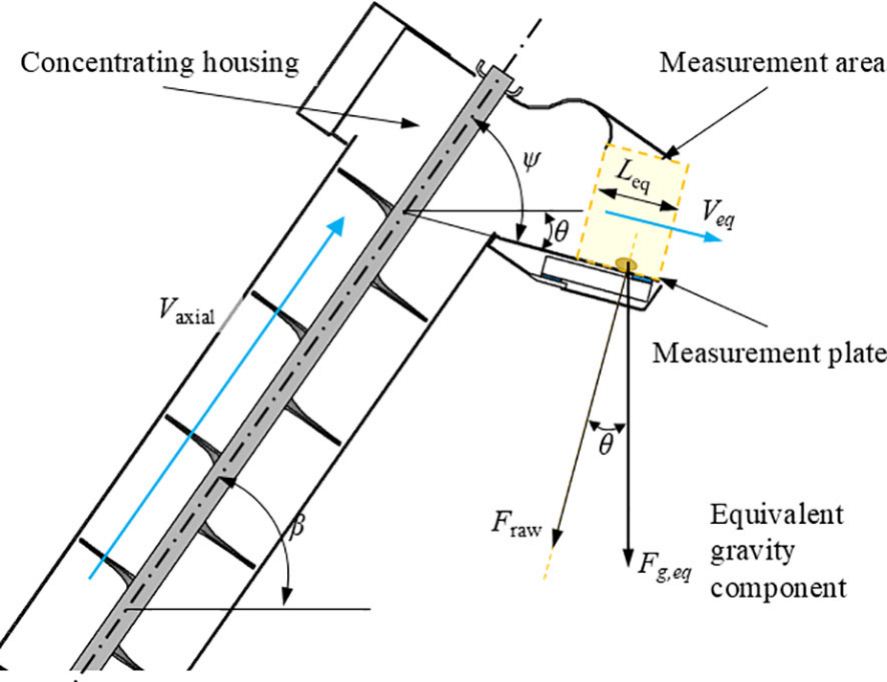
Schematic of dynamic weighing model for the inclined screw conveyor. *F_raw_* denotes the raw normal reaction force measured by the C-GMFS; *F_g,eq_* is the equivalent gravity component reconstructed along the vertical direction using the correction factor *k_F_*(*θ*).

In the inclined screw conveyor scenario, the converging and guided material flow is directed to the measuring plate through the collector structure, as illustrated in **Figure 7**. The raw signal output by the load cell is denoted as *F_raw_*(*t*), and the corresponding force decomposition relationships and parameter definitions are also shown in **Figure 7**. Since the mounting surface of the measuring plate forms a fixed angle *θ* with the gravity direction, an attitude correction coefficient *k_F_*(*θ*) was introduced to convert the sensor’s axial force into its equivalent component *F_g,eq_*(t):

(4) $$F_{g,eq}(t)=k_{F}(\theta )F_{raw}(t)$$

where, *θ* is determined by the screw conveyor shaft inclination angle *β* and the mounting angle *ψ* of the measuring plate, i.e., *θ*=*f*(*β*,*ψ*). After assembly, *θ* becomes a fixed value, making *k_F_*(*θ*) a constant. The theoretical value can be determined by the geometric relationship, and non-ideal factors such as the collector bend, friction, and assembly error are compensated via *in-situ* static calibration to ensure the consistency of the model within the engineering error range.

The complex structural configuration of the inclined screw conveyor’s discharge end makes it challenging to directly and stably characterize the equivalent average flow velocity *V_eq_*(*t*) via the screw conveyor shaft rotation speed, which is the core source of calculation error for existing dynamic weighing models in this scenario. To reduce the model’s dependence on empirical speed correction coefficients and additional external speed sensors, a residence time estimation method based on the time-domain features of the raw weighing signal from the C-GMFS was introduced in this study.

In practice, *τ_r_*(*t*) is estimated online from the time-domain footprints of successive discharge events extracted from the weighing signal. Let *F_lp_*(*t*) denote the low-pass filtered weighing signal. Following the event-detection chain used in the subsequent validity management, an envelope signal is constructed as shown in Equation 5:

(5) $$E(t)=LP_{f_{c,env}}(|\frac{dF_{lp}(t)}{d(t)}|)$$

where *LP_fc,env_*(·) denotes low-pass filtering with cutoff frequency *f*_c,env._ Candidate events are detected by applying a median–MAD robust threshold to *E*(*t*), as shown in Equation 6:

(6) $$E(t)>median(E)+k_{mad}\cdot \text{MAD}(E)$$

and detections separated by less than *t*_merge_ are merged as one event. For the *i*-th detected event, the start and end instants are denoted as *t*_s_^i^ and *t*_e_^i^, respectively, and the event width is defined as *w_i_*= *t*e i,− *t*_s_^i^. The event interval is defined as Δ*t_i_*=*t_s_^i^* + 1−*t*_s_^i^. Within each valid window, the equivalent residence time is estimated as *τ_r_*(*t*)=median(*w_i_*), while *CV_w_* and *CV*Δ*_t_* are computed from {*w_i_*} and {Δ*t_i_*} to assess event stability. Only when the event sequence satisfies the predefined validity constraints (e.g., sufficient event count and bounded dispersion) is *τ_r_*(*t*) accepted for online mass-flow reconstruction; otherwise, the output is withheld and the system can temporarily switch to the backup speed-based estimation module.

For the packet-wise grain flow at the discharge end of the inclined screw conveyor, the equivalent average residence time τr(t) of the grain in the closed measurement zone is defined as:

(7) $$\tau_{r}(t)=\frac{L_{eq}}{V_{eq}(t)}$$

Substituting Equations 4 and 7 into Equation 3, the core formula for mass flow calculation under inclined screw conveyor conditions is obtained as shown in Equation 8:

(8) $$q_{m}(t)=\frac{k_{F}(\theta )\cdot F_{raw}(t)}{g\cdot \tau_{r}(t)}$$

This model determines the instantaneous mass flow rate from the attitude-corrected weighing signal and the residence time of grain-flow packets, making it suitable for the pulsating and intermittent discharge characteristics at the inclined screw conveyor outlet. Unlike purely calibration-based approaches or fixed velocity-coefficient models, it preserves the physical interpretability of the mass-flow solution. To facilitate online estimation, a short-term quasi-steady assumption is adopted: within the time window in which a grain-flow packet passes through the measurement region, the accumulation–release process is approximated as quasi-steady, such that the equivalent residence time *τ_r_*(*t*) varies slowly within the window and the instantaneous equivalent mass in the measurement region can be represented by the posture-corrected equivalent gravity component. This assumption holds when the measurement boundary remains stable, bypass leakage is negligible, and the packet-wise flow pattern does not change abruptly within the window.

To facilitate the deployment of the model on the on-board embedded terminal, all constant terms were consolidated into a single calibration coefficient *K_F_*=*k_F_*(*θ*)/g, and the model was simplified as shown in Equation 9:

(9) $$q_{m}(t)=K_{F}\frac{F_{raw}(t)}{\tau_{r}(t)}$$

To adapt the simplified continuous-domain model to low-latency real-time computing on the on-board embedded terminal of the DW-ORMS, time-domain discretization was performed on the model in this study. For real-time recursive calculation with a fixed sampling period *T*, the instantaneous mass flow rate and cumulative net grain mass at the *k*-th sampling moment can be calculated using Equations 10 and 11:

(10) $$q_{m}(k)=K_{F}\frac{F_{raw}(k)}{\tau_{r}(k)}$$

(11) $$M(k)=M(k-1)+q_{m}(k)T$$

where *M*(*k*) is the cumulative net grain mass, kg; *T* is the sampling interval, s.

For engineering robustness, a backup rotational-speed-based estimation module was integrated into the DW-ORMS. It is activated only when the validity of residence-time estimation is insufficient and does not alter the formulation of the main residence-time-based model.

#### 2.4.2 Signal processing and data-handling strategy

To address the real-time embedded computing requirements under strong coupled vibration and intermittent discharge conditions at the discharge end of inclined screw conveyors, the DW-ORMS developed in this study employs a full processing chain comprising high-frequency data acquisition, low-latency preprocessing, event/validity management, online computation, multi-rate data output, and traceable storage. The signal and data flow direction is shown in **Figure 3**.

### 2.5 State-aware strategy and parameter fixation for online implementation

#### 2.5.1 Operational phase recognition

To solve the fuzzy boundary problem of the operational stages (no-load Δ*t*_0_, feeding increase Δ*t*_1_, stable operation Δ*t*_2_, grain cleaning and emptying Δ*t*_3_, and emptying back Δ*t*_4_) of the inclined screw conveyor, a rule-driven operation state recognition strategy was proposed. This strategy primarily utilized the low-frequency trend *F_lp_* and its slope *dF_lp_* of the weighing signal as key characteristic indicators, while integrating event sequence characteristics (event density, width stability, and *RMS_band_*) along with synchronously collected data on the screw conveyor’s rotational speed and forward speed. Adaptive partitioning of Δ*t*_0_-Δ*t*_4_ was achieved through a finite state machine (FSM) (**Figure 4**). Specifically, the system employed median-MAD-based hysteresis thresholds to construct “entry/exit” criteria. Within operation-related states, the robust threshold of *dF_lp_* was utilized to determine ascending, steady-state, and descending evolutions (corresponding to Δ*t*_1_-Δ*t*_3_). Δ*t*_0_ and Δ*t*_4_ were constrained by low load and low event density characteristics. Additionally, hold time *T_hold_*, minimum phase time *T_min_*, and gap-filling *T_gap_* were introduced to suppress boundary jitter and avoid phase fragmentation. **Figure 4** illustrates the overall logic of FSM phase recognition and validity determination (freezing/recovery update). The significance of the FSM lies not only in operational phase recognition itself, but also in converting phase recognition into update-authority management. By distinguishing reliable and unreliable operating segments, it prevents non-working or strongly disturbed periods from introducing irreversible bias into residence-time updating and cumulative mass integration.

#### 2.5.2 Validity assessment

On the basis of operational phase recognition, a validity assessment and gating mechanism was introduced to manage the reliability of online updates and cumulative mass integration. Phase recognition determines the current operating stage, whereas validity assessment determines whether the current output is acceptable for baseline updating and cumulative integration under the prevailing disturbance level. The event quality is defined by the stability of the impact events and the consistency of event widths, which are characterized by the coefficient of variation of the event intervals *CV*_Δ_*_t_* and the coefficient of variation of the event widths *CV_w_* respectively. The vibration severity is characterized by *RMS_band_* and the event rate *λ*. Based on these indicators, the confidence score *c*(*k*) is computed using Equation 12, which combines the event-quality indicator *Q*(*k*) and the vibration-severity indicator *V*(*k*), and is then compared with the threshold *c_min_* to determine the validity flag (valid-flag=1 if *c*(*k*)≥*c*_min_; otherwise, valid-flag=0):

(12) $$c(k)=\alpha_{1}Q(k)+\alpha_{2}V(k)$$

where *α*_1_ and *α*_2_ are weighting coefficients used to balance the contributions of *Q*(*k*) and *V*(*k*), respectively, subject to the normalization constraint *α*_1_+*α*_2_=1. In this study, *α*_1_ and *α*_2_ were selected based on preliminary field tuning to achieve a conservative validity decision, thereby reducing the risk of false-valid updates under severe disturbances. A brief sensitivity check indicated that the validity decision and the resulting cumulative integration remained stable within a reasonable range of *α*_1_ and *α*_2_. Specifically, *Q*(*k*) was normalized by *CV*_Δ_*_t_* and *CV_w_*, while *V*(*k*) was normalized by *RMS_band_* and *λ*. When valid-flag=0, the system froze the trusted update timing scale *τ_u_*, prohibited zero-point and baseline updates, and applied a hold/suppress strategy to the gated output mass flow $\hat{q}$*_m_*(*k*) to prevent irreversible deviations in cumulative integration caused by abnormal disturbance segments. Once the FSM entered the stable operation phase Δ*t*_2_ and *c*(*k*) continuously met the threshold for the required duration, the gating was automatically released, restoring online updates and normal output. Here, “hold” refers to retaining the last valid output before the invalid state was entered, while “suppress” means setting the output to zero.

#### 2.5.3 Parameter fixation and online implementation

The resulting thresholds and switching rules were fixed as deployable configuration parameters for online implementation, including the field-derived disturbance bounds, the event-detection parameters, the FSM timing constraints, and the trusted-update bounds. These parameters were written into the embedded system as runtime configurations, with the configuration version number synchronously stored in the data records to support offline traceability and reproducible comparisons.

#### 2.5.4 Threshold identification, validation, and fixation workflow

To improve traceability of the gating parameters, a four-step threshold identification and validation workflow was used. First, the field-derived intervals of [*f*_1_, *f*_2_], *RMS_band_*, *λ*, and *τ_u_* were identified from all field runs using global P10–P90 statistics and used as the admissible disturbance ranges for parameterized simulation. Second, these ranges were scanned in simulation to evaluate event recognisability, width consistency, trend fidelity, band-limited attenuation, and end-to-end latency, thereby defining the safe operating region of the online strategy. Third, the online decision thresholds for phase transitions were derived from median–MAD robust statistics of *F_lp_* and *dF_lp_*, while *T_hold_*, *T_gap_*, *T_min_*, and *t_merge_* were introduced as continuity constraints to suppress fragmented segmentation. Finally, the resulting parameter set was cross-checked on independent field runs and then fixed as deployable configuration parameters for the embedded implementation.

### 2.6 Implementation of the DW-ORMS

Based on the architecture described in Section 2.1, the hardware and software implementation of the DW-ORMS is detailed below. The system took the dynamic weighing observation data output by the C-GMFS as the main input, completed signal conditioning, high-frequency sampling, online preprocessing, and validity management, and calculated the instantaneous mass flow and cumulative net grain mass in real time according to the proposed model and strategy. The system synchronously integrated the screw conveyor’s rotational speed and GNSS data for operational logging and data synchronization, thereby establishing a closed-loop system featuring “inclined discharge end detection, real-time processing, instant output, and traceable storage”.

#### 2.6.1 Design of the C-GMFS for inclined discharge

**Figure 8:**
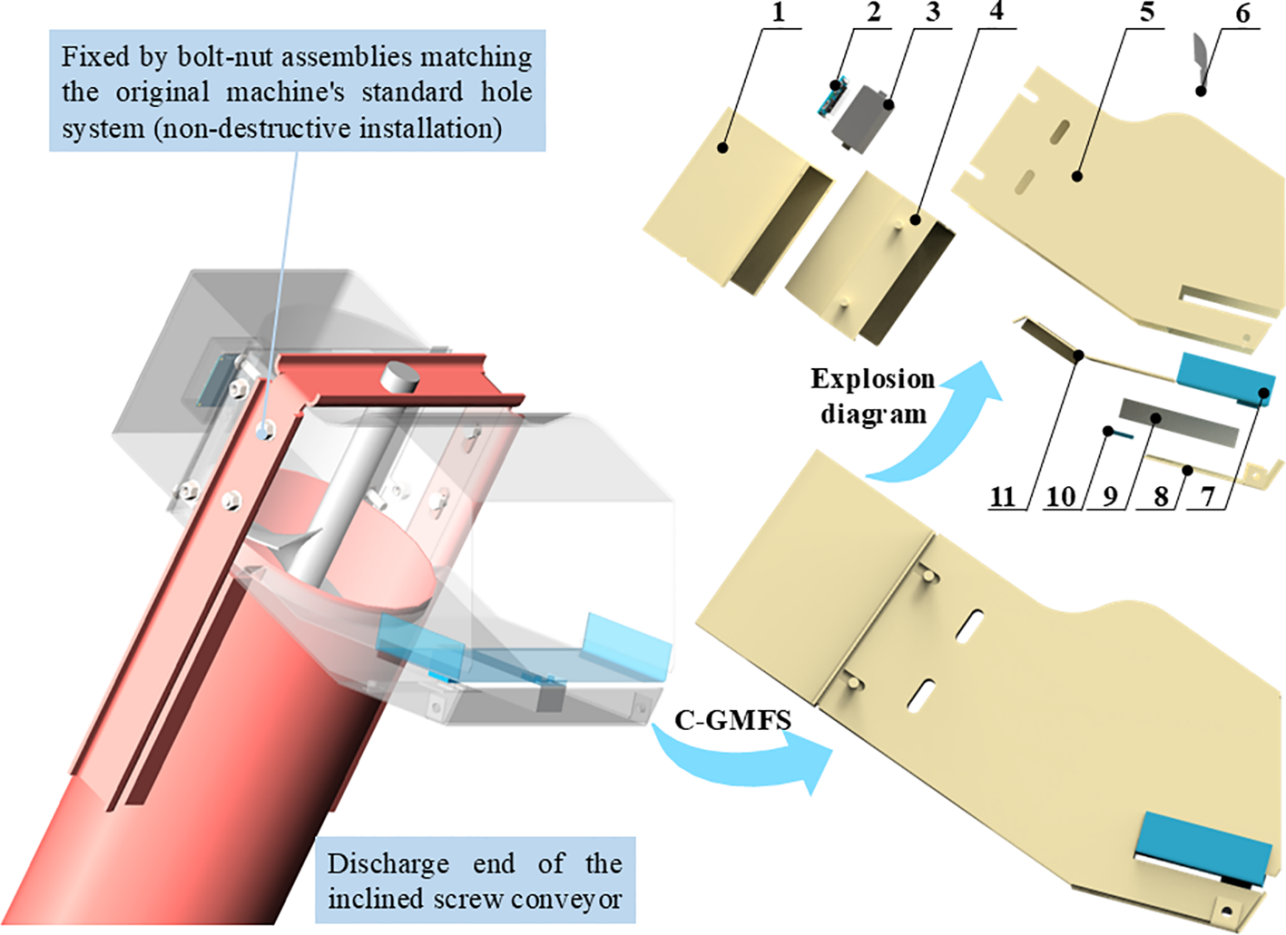
Structure and installation of the flow-collecting weighing-type grain mass flow sensor (C-GMFS) at the discharge end of the inclined screw conveyor, illustrating the key structural components that establish the measurement boundary, the mechanically decoupled force-transfer path, and the retrofit-oriented installation scheme. 1. Integrated hardware protective housing; 2. Signal acquisition and processing unit; 3. Acquisition and processing integrated box; 4. Shell sealing cover plate; 5. Flow collecting housing; 6. Flow guiding upper corner plate. 7. Measuring support plate. 8. Protective bottom plate; 9. Weighing unit; 10. Mounting stabilizing pad; 11. Flow guiding plate in flow collecting inner cavity.

Considering the limited installation space of the inclined discharge end and the combined effects of intermittent discharge and coupled vibration, the C-GMFS was designed in this study, as shown in **Figure 8**. The C-GMFS was mounted externally on the discharge end housing, allowing quick installation via the original bolt and nut connections. Non-invasive online monitoring of the clean grain flow was achieved without altering the existing conveyor channel structure or grain discharge process.

The C-GMFS was composed of a collector housing, a measuring plate assembly, a weighing unit, a mounting bracket, and a vibration isolation and limit assembly. The collector shell formed a continuous and smooth transition channel at the discharge end, which converged and redirected the scattered grain flow, establishing a relatively stable measurement boundary upstream of the measuring plate. By controlling the drop height and guiding surface, secondary impact and material retention were reduced. The measuring plate was arranged at the exit of the collector channel, and its fixed posture ensured that the geometric relationship between the direction of gravity and the sensitive axis of the sensor remained constant, thereby ensuring the repeatability of force decomposition and model parameters across different working conditions.

In the force transfer design, the measuring plate and single-point load cell formed the sole load-bearing path, with grain loads transmitted along the “measuring plate–weighing unit” route to the sensor’s sensitive direction. The weighing unit was connected to the main body via an independent mounting bracket, with vibration isolation and limit mechanisms installed at the connection point to mitigate the bypass coupling of housing structure loads and main body vibration on the weighing chain. A gap fit was adopted between the measuring plate and the housing, and flexible sealing was used to avoid the formation of rigid bypasses and non-target load input, thereby stabilizing the boundary conditions of dynamic weighing from the structural level. The sensor was protected and reinforced by a protective structure to meet the dustproof, impact-resistant, and durable requirements of field operations. These design features jointly improve installation compatibility and anti-interference capability, thereby enhancing the stability of dynamic weighing during practical harvesting operations.

#### 2.6.2 Development of the hardware system

The hardware architecture of the DW-ORMS mainly consists of a weighing sensor unit, an analog front-end conditioning and A/D sampling module, an embedded processing and communication module, and an auxiliary information interface. The raw weighing signal was processed by low-noise conditioning and an anti-aliasing filter, then input into the embedded processor at a high sampling rate to perform filtering, event/validity management, and mass flow model calculation. The screw conveyor’s rotational speed was measured by a Hall sensor and used for real-time calculation of speed-related parameters. GNSS signals were transmitted via the CAN bus to the system for trajectory recording and effective zone identification of harvesting strips. Essential operational status parameters were also accessed through the CAN bus, supporting phase determination and data annotation during operation. The embedded implementation was deployed on an STM32F407ZGT6 microcontroller (ARM Cortex-M4, 168 MHz, 1 MB Flash, 192 KB SRAM), which served as the core processing unit for high-frequency acquisition, online data handling, and real-time output management.

To achieve precise dynamic measurement of grain loads in the monitoring zone of the measuring plate, the weighing unit employed a single-point load cell (Model DJDD-130, Shanghai Di Jia Sensing Technology Co., Ltd, China), with a measurement range of 0 to 10 kg. The sensor features uniaxial vertical force response, with strong resistance to lateral components and torsional loads, making it suitable for dynamic weighing requirements under vibration conditions in screw conveyors. It has a maximum excitation voltage of 15 V (DC/AC), 2 mV/V output sensitivity, and 150% F.S. safe overload capacity. With an IP65 protection rating, it ensures long-term stable operation in field environments with dust, high humidity, and mechanical vibrations.

To obtain the rotational speed of the screw conveyor shaft and provide input for kinematic conversion, a Hall-type speed sensor (Model JKC8002C, Shanghai Ktgee Sensor Technology Co., Ltd, China) was selected as the speed measurement unit. The sensor operates on a non-contact magnetic pole induction principle to generate pulse signals, with a response time of ≤1 ms and an output frequency range of 0-10 kHz. It features an IP67 protection rating. During installation, the sensor was fixed on the shaft end bracket, and a permanent magnet was embedded in the shaft end to rotate synchronously with the screw conveyor shaft. When the magnetic pole passes through the sensor’s sensing area, the sensor outputs a square wave pulse. The acquisition end counts the pulse signal and converts it into a discrete rotational speed sequence, which serves as the input parameter for subsequent kinematic calculation and conveying speed characterization.

To ensure the temporal consistency of multi-source data, the system employed an embedded clock as a unified time reference. It uniformly timestamps weighing signals, rotational speed data, and GNSS information, then packages and outputs them within fixed cycles. The collected data can be used for test debugging and offline calculation via the serial port, and can also be integrated with the whole harvester system and displayed in real time through the CAN bus. The on-board DC power supply, after isolation and filtering, powers both analog and digital circuits. It is equipped with overvoltage, reverse connection, and surge protection circuits to withstand the complex vibration and electromagnetic environment in the field, ensuring stable system operation.

In practical field deployment, calibration drift may arise from temperature variation, vibration overload, and mounting looseness. Temperature variation may affect the zero offset and sensitivity of the load cell, while repeated vibration and shock loading may disturb the effective force-transfer path or introduce zero-point fluctuation. Mounting looseness may further change the mechanical boundary condition of dynamic weighing during long-term operation. In the present system, these effects were mitigated through a single force-transfer path, an independent mounting bracket with vibration isolation and limit mechanisms, flexible sealing and protective structures, and zero/baseline management in the online processing chain. Nevertheless, long-term deployment still requires periodic inspection of mounting stiffness, sealing integrity, wiring reliability, and static calibration stability. Dedicated long-term drift validation under extended field use will be further investigated in future work.

#### 2.6.3 Development of the software system

**Figure 9:**
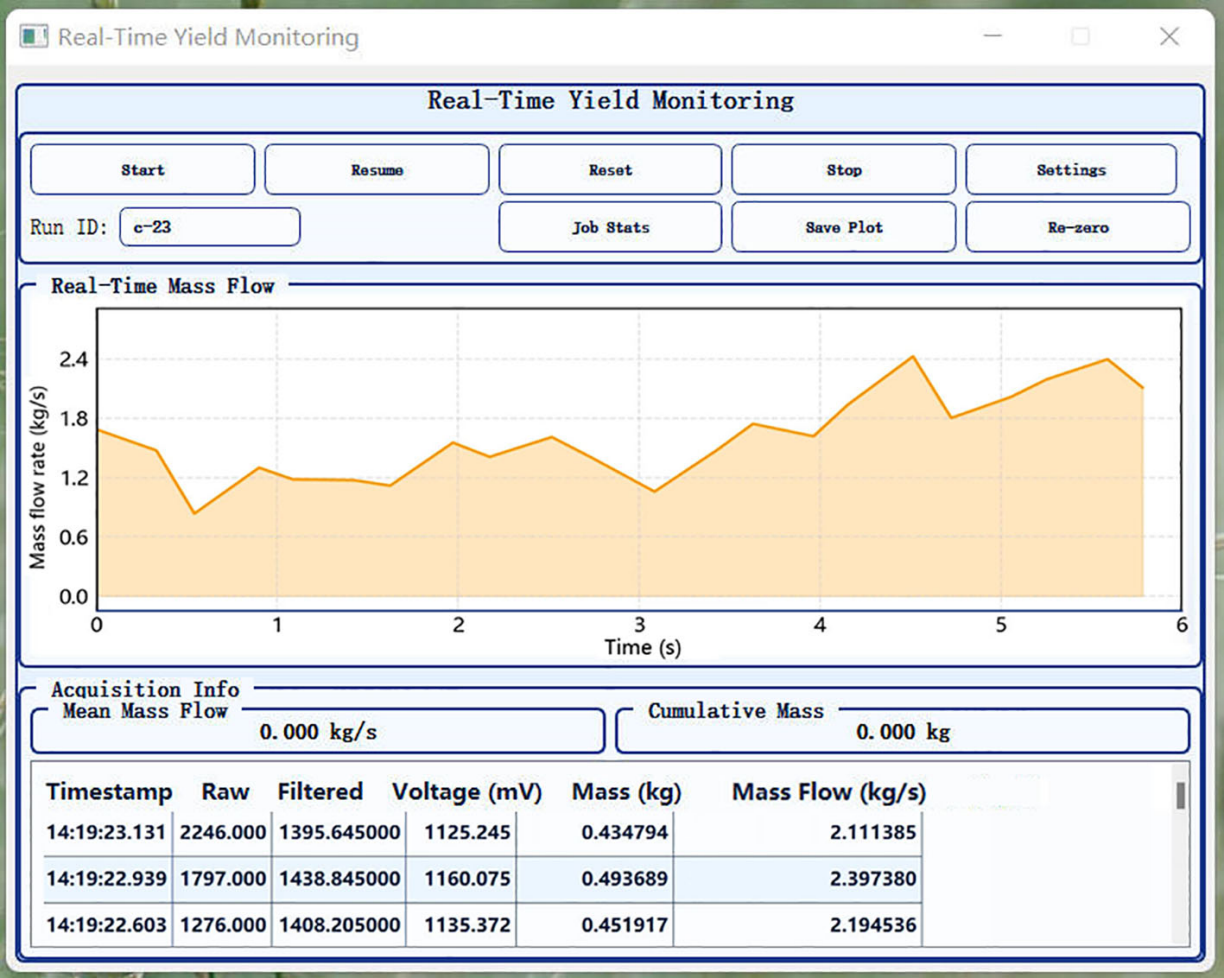
Upper-computer software interface of the DW-ORMS for parameter configuration, real-time state monitoring, result visualization, and traceable data management during field operation and offline replay.

The on-board software consisted of embedded firmware and host computer software. The firmware was responsible for multi-channel sampling, online filtering, FSM execution and validity judgment, mass flow and cumulative mass calculation, and output key monitoring quantities, as well as necessary raw and intermediate quantities, at the set frequency to support traceability analysis. The host computer software (**Figure 9**) handled parameter configuration, status monitoring, result visualization, and data management. It supported HDF5 format data storage, providing a unified entry point for subsequent offline traceability.

In the present system, the weighing signal from the load cell was acquired through the ADC and then processed on the embedded platform through digital low-pass filtering, phase gating, Alpha–Beta estimation, and grain-mass-flow calculation. To quantify the timing performance of the current implementation, an oscilloscope-based timing test was conducted. The input excitation signal was used as the trigger reference, and a GPIO pin was toggled when the updated grain-mass-flow output became available. The time interval between the two signals was taken as the practical end-to-end latency of the current implementation.

## 3 Results and discussion

The simulation results were used to evaluate the design rationale and robustness of the proposed state-aware strategy, whereas the overall monitoring performance and engineering effectiveness of the system were assessed solely through field-operation trials.

### 3.1 Field-disturbance characteristics

To support the subsequent simulation design and threshold fixation, the field-derived disturbance characteristics are first presented.

**Figure 10:**
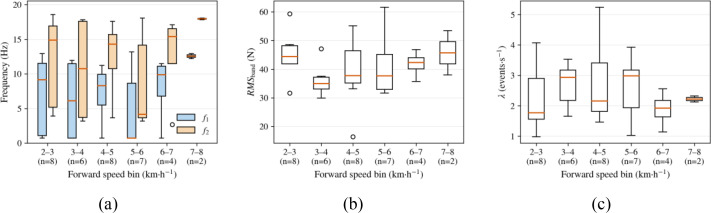
Statistical results of field disturbances grouped by forward speed: **(a)** dominant band edges [*f*_1_,*f*_2_] ; **(b)** band-limited disturbance intensity *RMS_band_*; **(c)** impact density (event rate) λ.

**Figure 10** summarizes the field-disturbance statistics grouped by forward speed. As shown in **Figure 10a**, the dominant disturbance band shifted upward overall with increasing speed. By contrast, **Figures 10b, c** show that *RMS_band_* and impact density *λ* varied in a condition-dependent manner, with relatively higher levels observed in some medium-to-high speed intervals.

### 3.2 Simulation-based evaluation under varying vibration intensity and impact density

To quantify how different field disturbance conditions affect the characteristics of the weighing signal and the estimation of residence time, and to determine a conservative operating region for the online gating rules, a disturbance scanning and performance evaluation were conducted based on a parameterized disturbance model. The simulation parameters were specified according to field statistics, and the scanning dimensions included the band-limited vibration intensity *RMS_band_* and the impact density *λ*. For each disturbance combination, disturbance realizations were generated repeatedly to evaluate the validity of state/event recognition, the statistical properties of event time scales, and the filtering/reconstruction performance.

To directly delineate, on the scanning plane, an identifiable window in which event time scales are estimable and phases are recognizable, the validity decision in simulation was simplified into operational criteria based on event quantity and width consistency (event_count≥*N_min_* and *CV_w_*≤*CV_w,max_*), and a valid-rate map was generated accordingly. Here, event_count denotes the number of valid events recognised in a single simulation run, *N*_min_ is the minimum threshold of events required for reliable statistics, *CV_w_* is the coefficient of variation of event widths, and *CV_w,max_* is the maximum allowable variability. These criteria indicate whether a given disturbance condition still allows the system to detect enough events with sufficiently stable time scales for residence-time estimation. Based on these criteria, the valid rate was defined as the mean value of the valid-flag over repeated simulation runs under the same disturbance combination.

It should be noted that, in simulation, valid-flag served only as a proxy indicator of event quality for quantifying identifiability; in the online implementation, the confidence *c*(*k*) was still computed by fusing event-quality measures with the disturbance proxies *RMS_band_* and *λ*, and the gating decision was executed accordingly.

**Figure 11:**
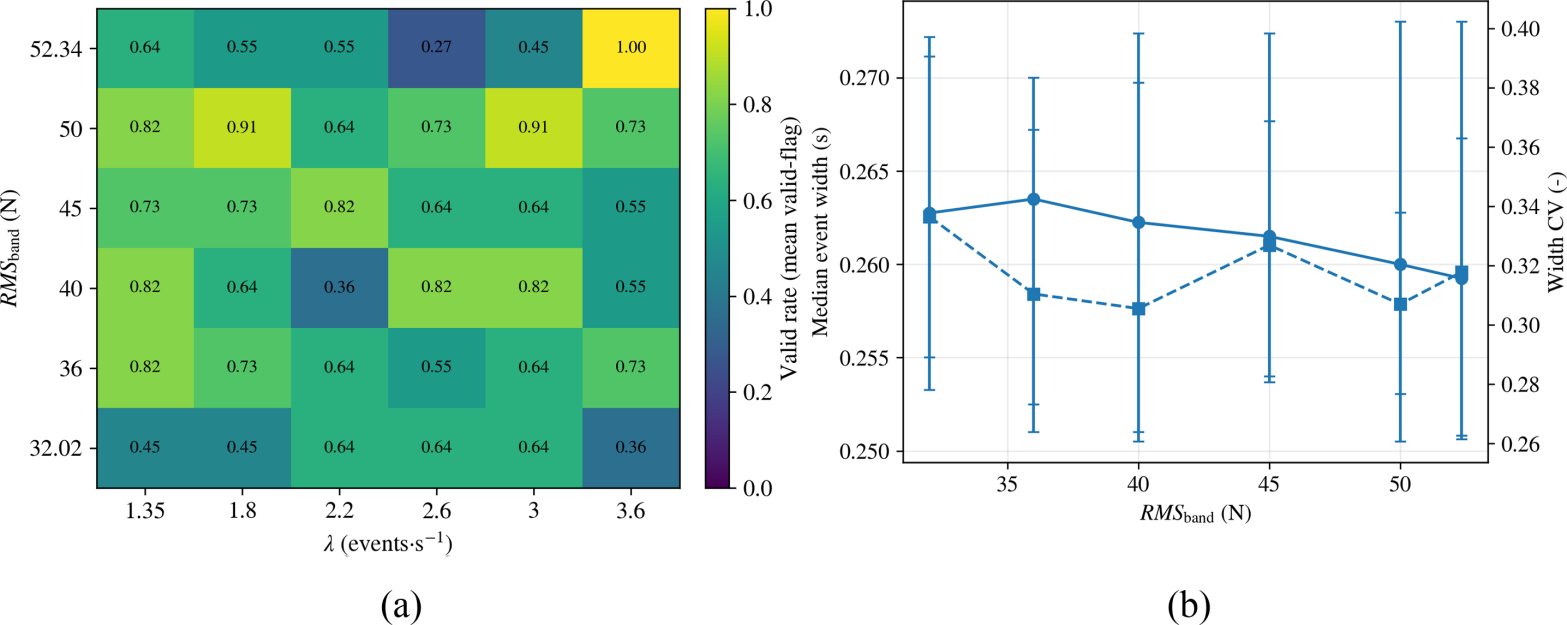
Simulation-based identifiability and event-scale statistics under parameterized disturbance scanning: **(a)** validity map in the disturbance-scanning space; **(b)** event-width statistics versus disturbance intensity.

As shown in **Figure 11a**, among all 36 perturbation combination grid points, the average valid rate was 0.656 with a median of 0.640. Specifically, 25.0% of grid points achieved high validity (valid rate≥0.80), 58.3% reached moderate validity (0.50≤valid rate<0.80), and only 16.7% fell into low validity (valid rate<0.50). Therefore, the online gating adopted a conservative “avoid-zone” principle, freezing τ updates when valid-flag=0. Additionally, 69.4% of grid points maintained valid rates≥0.64, indicating the proposed method retained robust state/event recognition capability under most scanning conditions. Notably, the parameter space exhibited a local low-efficiency valley at 0.27, corresponding to *RMS_band_* = 52.34 N and *λ* = 2.6 events·s^−^¹. This suggests that superimposed continuous vibration and dense impacts may cause event-footprint overlap and envelope distortion, thereby compromising recognition stability. The “worst-coupling point” was used to validate the safety boundary of the discernible window and to guide conservative parameter configuration.

As shown in **Figure 11b**, within the scanned *RMS_band_* range, the median event width fluctuated within a narrow range of approximately 0.260–0.263 s, with a corresponding coefficient of variation (CV) of about 0.31–0.34. This shows that the time scale of events has good statistical stability in the identifiable region, which can provide a reliable basis for the online estimation of *τ*. Taken together with **Figure 11a**, the occurrence of low-efficiency points is not due to the overall divergence of event time scales, but is more likely caused by event-footprint overlap, envelope distortion, and mismatched gating criteria under local perturbation coupling. Accordingly, the low-efficiency region was treated as an unreliable update zone in online implementation, where residence-time updating was frozen until the disturbance level returned to an identifiable range. This strategy helps prevent strongly nonstationary disturbances from introducing irreversible bias into cumulative integration.

**Figure 12:**
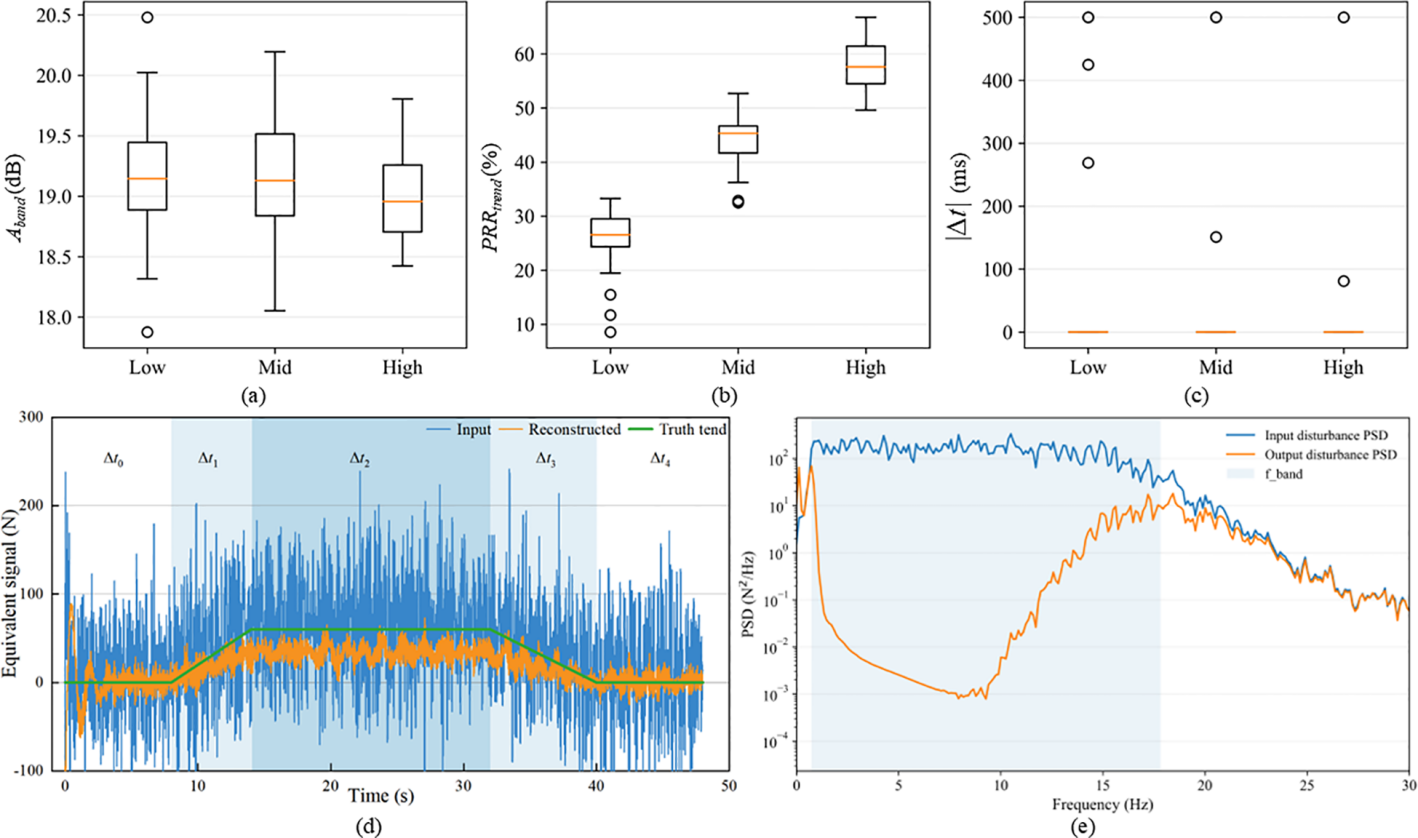
Simulation performance and representative examples across disturbance levels: **(a)** band-limited attenuation *A_band_*; **(b)** trend fidelity *PRR_trend_*; **(c)** end-to-end latency |Δ*t*|; **(d)** time-domain reconstruction under a high-disturbance condition; **(e)** PSD comparison under a high-disturbance condition.

After delineating the discernible window, the proposed method was evaluated across disturbance levels from two perspectives: statistical performance and a representative example. The statistical results in **Figures 12a–c** show that the proposed method maintained stable in-band attenuation, acceptable trend fidelity, and low end-to-end latency across different disturbance levels. The trend-preservation metric *PRR_trend_* stayed within an acceptable range as disturbances increased, and its distribution became more concentrated under moderate-to-high disturbances, suggesting that the reconstructed signal still retained the true trend under strong perturbations. The median end-to-end delay |Δ*t*| was close to zero for all three conditions, with only a few outliers, meeting the real-time requirement for online processing.

**Figure 12d** further illustrates the time-domain reconstruction under high disturbance. The raw input exhibited large-amplitude, strongly non-stationary fluctuations due to coupled vibration and impacts, whereas the reconstructed output closely followed the true trend during feeding increase (Δ*t*_1_), steady operation (Δ*t*_2_), and grain cleaning/emptying (Δ*t*_3_), without evident baseline drift or phase misalignment. For this case, *A_band_* = 19.19 dB, *PRR_trend_* = 61.7%, *RMSE_trend_* = 20.12 N, and |Δ*t*|=0.00 ms, supporting the capability to jointly achieve disturbance suppression, trend tracking and low-latency response under severe perturbations.

Frequency-domain evidence is consistent with these observations (**Figure 12e**). Within the operating band *f_band_*=[0.73,17.82] Hz, the output disturbance spectrum was markedly lower than the input, confirming effective suppression of structural vibration and impact-vibration coupling within the band.

In addition to the simulation-based latency assessment, an oscilloscope-based timing test on the embedded implementation yielded a worst observed delay of 97.52 ms. This result reflects the overall output latency of the onboard processing chain, including the causal filtering and phase-gating delays, and confirms the practical real-time feasibility of the current implementation.

### 3.3 Field harvesting results of the DW-ORMS

#### 3.3.1 Overview of field operating conditions and datasets

**Figure 13:**
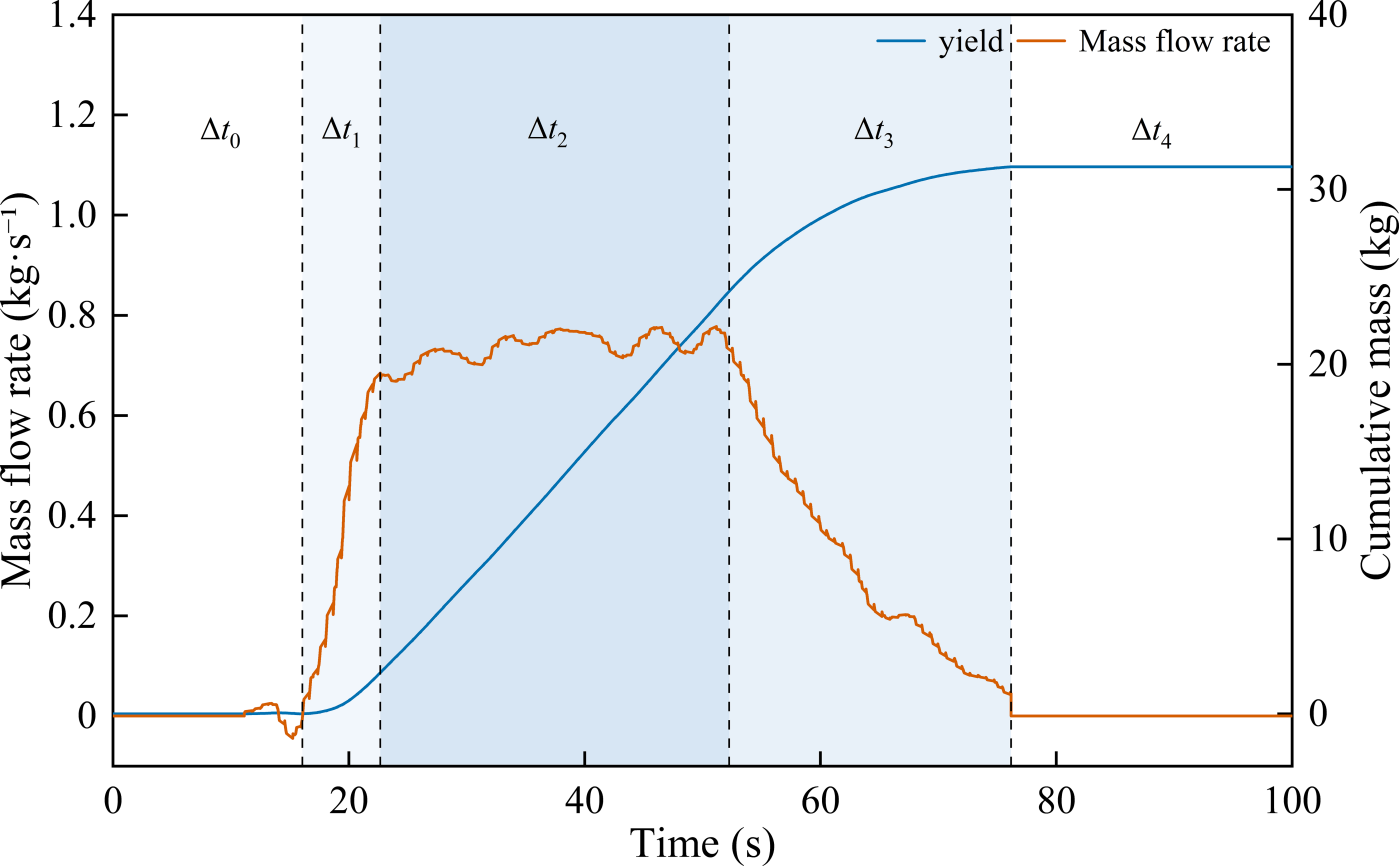
Mass flow and cumulative mass variation during typical field operation. The left axis shows the instantaneous grain mass flow rate (kg·s^−^¹), and the right axis shows the cumulative mass (kg) obtained by integrating the gated flow-rate output within the valid window. The shaded (or marked) intervals indicate the validity-gated periods used for cumulative mass calculation, while invalid periods are excluded to reduce the influence of vibration and discharge transients.

To verify the output stability and cumulative-mass consistency of the DW-ORMS under non-steady field conditions, field harvesting experiments were conducted. The test covered the range of 2-8 km·h^−^¹ forward speed, and 36 strip data were collected and analyzed, one strip was excluded because of abnormal data, and 35 strips were kept for performance evaluation. The typical field operation process is shown in **Figure 13**.

As illustrated in **Figure 13**, the DW-ORMS effectively tracked and responded to variations across different operational phases. During the feed increase (Δ*t*_1_) and cleaning discharge (Δ*t*_3_) stages, the system maintained continuous instantaneous mass flow within the gated effective window, preventing non-steady disturbances from entering the cumulative integration process. Consequently, the cumulative mass curve exhibited no spurious accumulation caused by baseline drift or false triggering. During the stable harvesting stage (Δ*t*_2_), the instantaneous flow fluctuated with feeding variations, while the cumulative mass curve increased monotonically and nearly linearly. These results demonstrate the system’s dynamic tracking capability and reliable integration performance throughout the entire harvesting process.

#### 3.3.2 Overall mass closure performance across all runs

**Figure 14:**
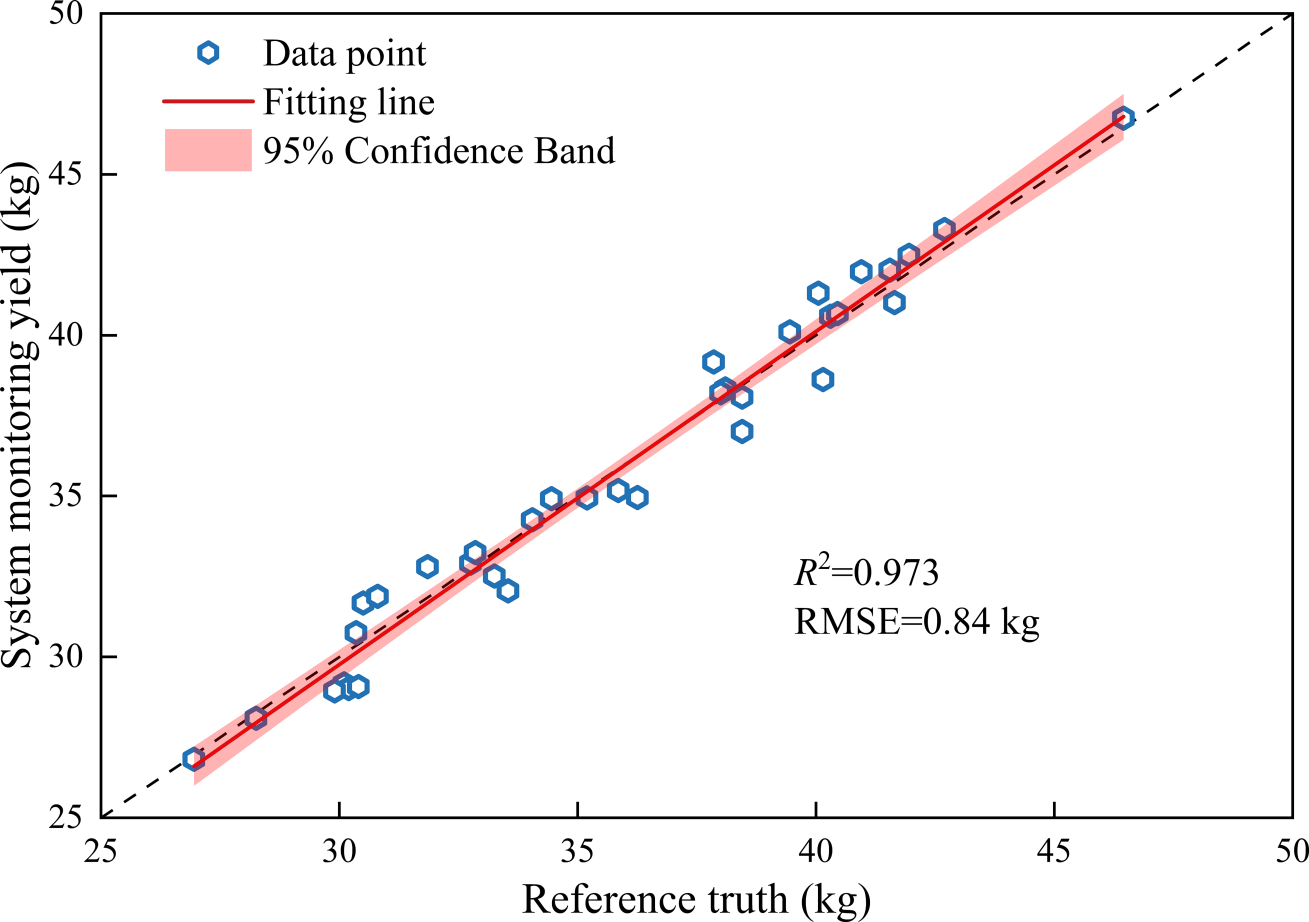
Strip-scale mass-closure validation. Scatter plot of system-estimated cumulative mass versus manually weighed reference mass for each strip; the dashed line denotes the 1:1 line, and the solid line is the linear fit with the 95% confidence band. The goodness of fit (*R*^2^) and RMSE are reported in the panel.

**Figure 14** validates the strip-scale yield closure by comparing the system-monitored yield with the manually weighed reference mass.

Excellent agreement was observed between the cumulative strip mass monitored by the C-GMFS and the manual weighing reference values, with scattered points densely distributed near the 1:1 line and no systematic overestimation or underestimation tendency. Linear regression yielded *R*^2^ = 0.973 and *RMSE* = 0.84 kg (**Figure 14**). These results demonstrate that the proposed C-GMFS measurement chain, together with the state-aware gating strategy, effectively suppresses the contamination of non-stationary disturbances on cumulative integration, achieving stable closure at the strip scale.

To further evaluate the impact of feed rate (inferred from forward speed) on closure performance, the closure error (CE, %) was categorized into three groups based on forward speed: low (2–4 km·h^−1^), medium (4–6 km·h^−1^), and high (6-8 km·h^−1^). **Table 3** presents the mean values, 95% confidence intervals, and robust statistics for each group. The mean CE values for the three groups were 0.37%, –0.09%, and –1.52%, respectively—all centered near zero and exhibiting no monotonic variation with increasing speed. The dispersion metrics (IQR, MAE) in the high-speed group showed relatively higher values, indicating that intensified disturbances amplify error fluctuations. At higher forward speeds, intensified structural vibration and more frequent discharge impacts reduce the separability of successive events in the weighing signal, which increases the dispersion of *CV*_Δ_*_t_* and *CV_w_* and consequently amplifies the fluctuation of *τ_r_* estimates. Meanwhile, the effective valid-update window becomes shorter because invalid segments are triggered more frequently under elevated disturbance levels. These factors together lead to the larger error dispersion observed in the high-speed group. It should be noted that the high-speed group contained only 6 samples, which may limit the statistical robustness of the corresponding conclusions. Therefore, the results for this group should be interpreted with caution. Nevertheless, the CE remained within ≤5% across all 35 tests, satisfying the engineering requirement of CE ≤ 5% (**Table 3**). These findings further verify the stability of the DW-ORMS across the 2–8 km·h^−1^ operating range in terms of strip-scale cumulative mass consistency and adaptability.

**Table 3:** Strip-level closure error grouped by forward speed.

Speed group (km·h^-1^)	N	Mean speed (km·h^-1^)	Mean CE (%)	95% CI of mean (±%)	Median CE (%)	IQR (%)	MAE (%)
Low (2–4)	14	2.65	0.37	0.42	0.59	0.93	0.75
Mid (4–6)	15	4.75	-0.09	1.50	1.35	4.76	2.48
High (≥6)	6	6.62	-1.52	4.25	-3.86	5.94	3.99

CE denotes strip-level closure error (%). “95% CI (±%)” is the half-width of the 95% confidence interval of the mean. Speed bins are defined as Low: *v*∈[2,4) km·h^−^¹; Mid: *v*∈[4,6) km·h^−^¹; High: *v*∈[6, 8] km·h^−^¹.

#### 3.3.3 Effect of forward speed on strip-level mass-closure performance

**Figure 15:**
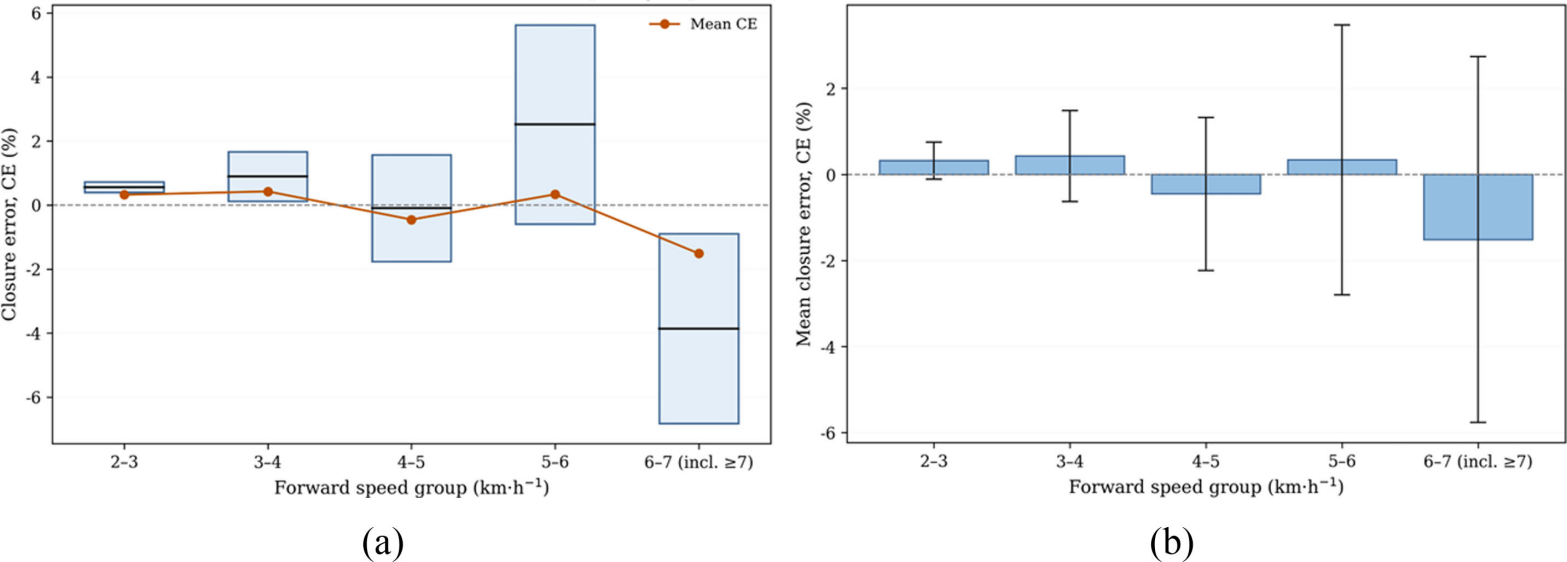
Effect of forward speed on strip-level mass-closure accuracy. **(a)** Distribution of strip-level closure error (CE) across forward-speed groups; **(b)** Mean CE with 95% confidence intervals. The CE values were grouped according to the average forward speed of each strip run within the range of 2–8 km·h^−^¹. In panel **(b)**, markers represent the group mean, and error bars denote the 95% confidence interval.

To further elucidate the distribution characteristics of closure error with respect to forward speed, **Figure 15** presents the CE statistical results across finer speed intervals, complementing the robust statistical comparisons for the three aggregated groups provided in **Table 3**.

**Figure 15a** shows the distribution of CE across finer forward-speed groups, whereas **Figure 15b** summarizes the corresponding group means and 95% confidence intervals. The grouped results show that the error remained centered near zero throughout the tested speed range, indicating that the proposed method did not introduce a systematic speed-dependent bias into strip-level cumulative mass closure within the tested operating range. Meanwhile, the dispersion increased in the highest speed interval, which is consistent with intensified vibration-impact coupling under high-load conditions.

#### 3.3.4 Comparison with a backup speed-based module

To provide a baseline reference, the proposed method was further compared against the backup rotational speed-based estimation module using the same 35 valid strip runs. The comparison was performed under the same evaluation protocol for strip-level cumulative mass closure. The results are summarized in **Table 4**. Compared with the backup rotational speed-based estimation module, the proposed residence-time-based method achieved lower closure deviation, lower cumulative mass error, and better overall consistency, further demonstrating the practical advantage of introducing residence-time estimation and state-aware gating under the inclined discharge conditions.

**Table 4:** Comparison between the proposed method and the backup rotational speed-based module.

Method	Mean CE (%)	MAE (%)	RMSE (kg)	*R* ^2^
Proposed method	-0.15	2.05	0.84	0.973
Rotational-speed-based module	0.45	3.72	1.56	0.917

## 4 Conclusion

To address the challenges of strong composite vibration interference and non-steady flow evolution in online yield monitoring of combine harvesters, this study developed a flow-collecting weighing-type grain mass flow sensor (C-GMFS), proposed a state-aware gating strategy based on parameterized disturbance simulation, and constructed a real-time monitoring system (DW-ORMS). The system performance was systematically validated through field trials. The main conclusions are as follows:

1. The developed C-GMFS provided a stable and physically traceable weighing basis for the inclined discharge end by establishing a reproducible measurement boundary and a mechanically decoupled force-transfer path under confined and vibration-prone conditions.
2. A closed-loop processing framework of “field statistics—parametric simulation—parameter solidification” was established to support operational state recognition and validity judgment under non-steady conditions. Simulation results showed that the proposed strategy achieved significant band-limited disturbance suppression (*A_band_*≥17.73 dB), while maintaining high trend fidelity (*PRR_trend_*≥41.73%) and low processing delay (|Δt|≤141.2 ms).
3. Field trial results demonstrated that the DW-ORMS achieved consistent strip-level mass-closure performance across the working speed range of 2–8 km·h^−^¹, with a cumulative closure error within ±5% (N = 35), indicating its practical applicability for on-board yield monitoring under the tested conditions.

Overall, this study establishes an engineering-feasible pathway for high-confidence on-board yield monitoring of combine harvesters. The non-destructive retrofit design and traceable strip-level validation of the DW-ORMS also indicate its potential for scalable upgrading of conventional combine harvesters in precision-agriculture applications. Future work will further examine long-term drift, cross-crop adaptability, and performance robustness under broader machine configurations and field environments.

## Data Availability

The raw data supporting the conclusions of this article will be made available by the authors, without undue reservation.
